# Hijacked: Co-option of host behavior by entomophthoralean fungi

**DOI:** 10.1371/journal.ppat.1006274

**Published:** 2017-05-04

**Authors:** Andrii P. Gryganskyi, Bradley A. Mullens, Michael T. Gajdeczka, Stephen A. Rehner, Rytas Vilgalys, Ann E. Hajek

**Affiliations:** 1 Department of Biology, Duke University, Durham, North Carolina, United States of America; 2 Department of Entomology, University of California Riverside, Riverside, California, United States of America; 3 Systematic Mycology and Microbiology Laboratory, USDA-ARS, Beltsville, Maryland, United States of America; 4 Department of Entomology, Cornell University, Ithaca, New York, United States of America; McGill University, CANADA

## Introduction

**Fig 1:**
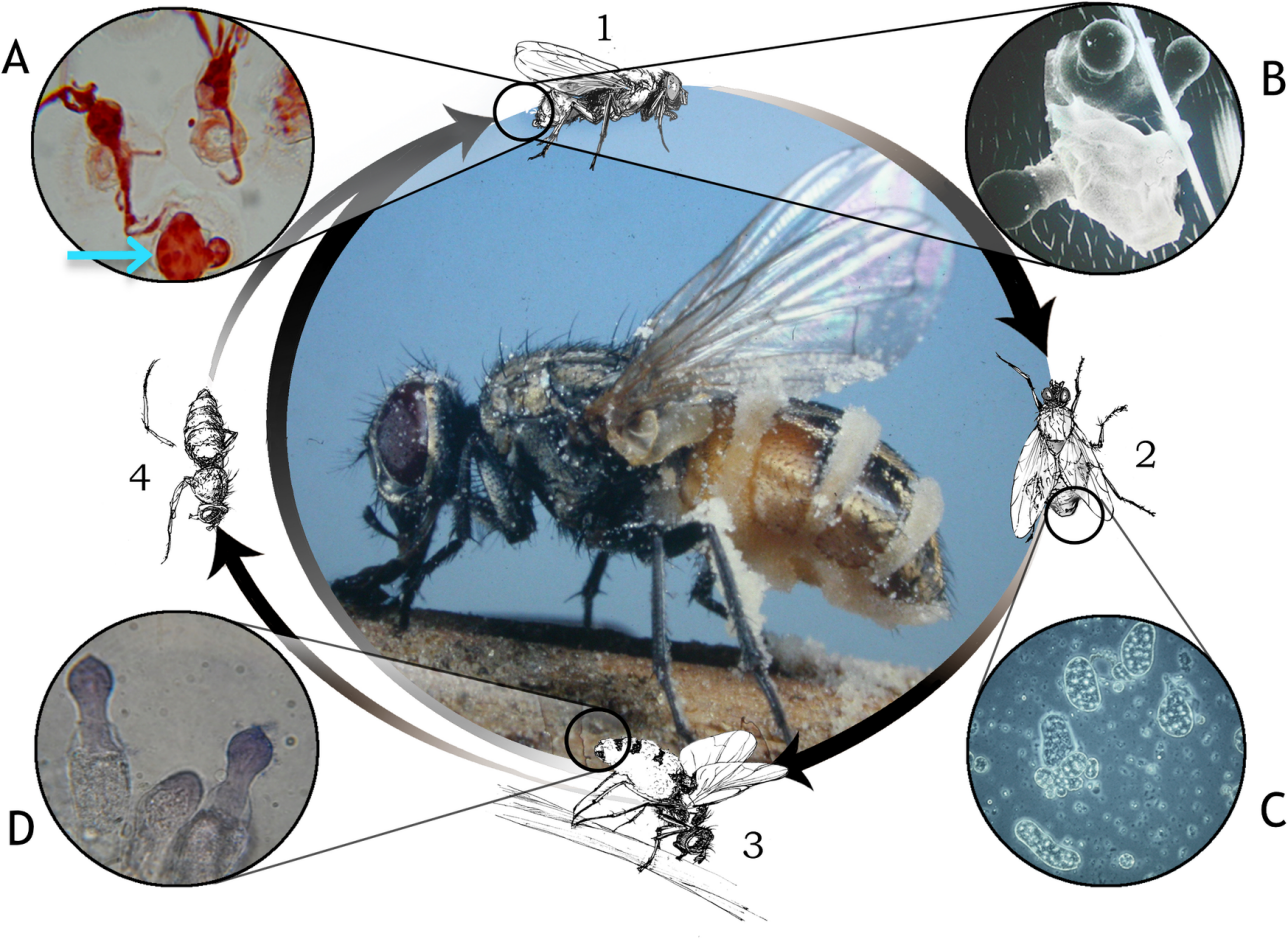
Life cycle of *Entomophthora muscae* infecting a muscoid fly. Beginning of infection (1, top). Germination of multinucleate primary infectious conidia on multiple locations on the host surface, producing hyphae and secondary conidium (A) or multiple secondary conidia (B). Infected fly in an intermediate stage of incubation (2) with interior hyphal bodies in insect blood and tissues within the body cavity (C). Death grip of actively sporulating cadaver (3); fungal conidiophores with young primary conidia emerging from membranous areas of host abdominal cuticle (D). Mummified fly cadaver (4) as a potential reservoir for overwintering resting spores. Center: House fly after most conidial discharge is complete. Note lifelike stance, with extended wings, legs, and mouthparts and sticky conidia adhering to legs, wings, and substrate. Photographs: Bradley Mullens, Max Badgley, and Andrii Gryganskyi; drawings by Danni Wei, student artist at UC Riverside.

Zombie-like fly, a fly behaviorally reprogrammed by its fungal parasite, makes its final journey up a blade of grass, just as the setting sun disappears. By early morning, the fly will have been dead for hours, its swollen, fungus-striped cadaver perched atop the dew-flecked grass and its wings spread as though ready to take flight. Its proboscis is fully extended and attached to the grass, as if this vegetation were the most delectable item of its last supper. Even now the cadaver attracts new fly victims, particularly love-inspired males mesmerized by sexual attraction to these macabre fungus-filled flies [1, 2]. For much of the night, the fungus has been producing and forcibly expelling a succession of sticky conidia (asexual spores). These conidia liberally shower the environment within a few centimeters of the fly cadaver, travelling a distance ~1,000–1,500 times greater than the diameters of the spores themselves. Infective secondary spores made by the primary conidia shot from the cadavers also fill the air and seed surrounding surfaces with biological bullets of death. And now, the fungus is poised to intercept new victims and repeat the infection cycle (**Fig 1**).

This dramatization describes fungal epizootics caused by *Entomophthora muscae*, which infects over 20 fly species from several families. Certain aspects of this disease (e.g., the climbing behavior of critically ill hosts) [3] are typical for quite a few fungal pathogens of arthropods, of which over 750 species are known throughout most major fungal and fungi-like lineages [4, 5]. Many of these arthropod pathogenic fungi belong to the phylum Entomophthoromycotina and most of the remainder are ascomycetes. Together, these fungi infect members of nearly all of the large arthropod taxonomic groups. The best characterized and most commonly witnessed epizootics are caused by entomophthoralean species infecting flies, aphids, grasshoppers, caterpillars, mosquitoes, cicadas, and mites [6].

## How do entomophthoralean fungi infect and kill insects?

Infection starts with the germination of a conidium. Adhesive conidia germinate on the surface of the insect cuticle, generating infectious hyphae, which penetrate multiple cuticle layers by combining digestive enzymes and mechanical pressure from hyphal turgor [7]. The infective fungus then grows either as hyphae, hyphal bodies, or protoplasts that proliferate inside of insect hosts, with the type of cells in a host related to the pathogen species. The cells absorb nutrients from their host and proliferate by budding or simple division. Hyphal bodies are short, often polymorphic, yeast-like mycelial fragments, while protoplasts lack sugar-rich cell walls. Protoplasts of the caterpillar pathogen *Entomophaga aulicae* continuously inhibit fungal glucan and chitin synthase activities and, by doing so, cell walls are not formed, and insect host hemocytes that normally protect hosts do not recognize these foreign invaders [8]. Pathogens growing as protoplasts acquire cell walls before host death, when the fungus gets ready to sporulate.

In order to expose conidia to the environment, the conidiophores develop a rigid cell wall capable of penetrating the insect cuticle, this time from the inside out. The conidia produced become airborne and can usually be detected near or below the insect cadavers, as well as being readily recovered from the air [9]. Primary conidia that don’t intercept a suitable host germinate and produce smaller secondary conidia. In many entomophthoralean species, secondary conidia also differ morphologically from the primary conidia, and in some cases they are the main infection stage. In the absence of reaching a host, subsequent tertiary or even quaternary generations of conidia might develop, either until a host is encountered or until the energy and resources required for spore proliferation and germination are exhausted.

## Who’s really in control—The fungus or its host?

As infection proceeds, insects lose vital functions. They slow down, eat less, stop laying eggs, or deposit eggs in inappropriate places [3, 10, 11]. An infected host may seek warm, dry places (behavioral fever) within the early and middle stages of the infection cycle (i.e., incubation days 2–4). This behavior appears to increase the host’s chances of overcoming the infection or at least delay disease progression [12]. Behavioral fever in infected house flies has been reported using thermal gradients in the lab [13] and in the field [14]. In the final 1–2 days of infection, a substantial shift in host behavior occurs. The fungus assumes control and co-opts flies to seek out cooler locations advantageous to the pathogen. As in our dramatization above, the top of a blade of grass at sunset is often cooler than the sun-warmed soil. Notably, behavioral fever is a symptom that appears in many poikilothermic hosts infected by a variety of pathogens, including some non-fungi [15].

As the host nears death, relatively elevated positions are favorable to spore dispersal. In or on buildings, this may be the tops of windows or walls. In open areas, locations typically are 5–80 cm above the ground on the tops of plants or other structures [15–18]. This climbing pattern for hosts can be observed also in cages with no temperature gradient; shortly before death, flies will scale their cage and expire near the top [10, 16].

The progressive infection pattern spares critical tissues such as flight muscles or nerves until the end, allowing hosts to move around and obtain nutrition from 1.5 days to several years, but usually for about a week [4]. In a few bizarre situations, the host even remains alive during fungal sporulation. This is characteristic of *Delia* flies infected by *Strongwellsea castrans* or *Magicicad*a spp. infected by *Massospora* spp. In these cases, the insect abdomen is co-opted, sterilizing the hosts [19, 20] and turning them into flying spore dispersers.

As the host nears death, the fungal pathogen changes the host behavior to facilitate its attachment to a substrate (the so-called “death grip”). Tarsal claws at the distal end of the legs provide a firm attachment as the fly’s legs contract. Often the fungus grows from the extended proboscis, providing an even stronger attachment. Many entomophthoralean fungi grow from and attach these dying or dead insects to a substrate so that cadavers are anchored in a specific location [3].

The biochemical and physiological mechanisms of host behaviors manipulated by fungal pathogens are still not understood. Perhaps the fungus secretes molecules (e.g., eicosanoids) functionally similar to those produced by the insect itself. These molecules might promote taxis behaviors (e.g., in relation to temperature, humidity, sunlight, or gravity) or specific muscle-related functions such as wing or proboscis extension. Recent studies have shed some light on the molecular mechanisms of entomophthoralean pathogenesis [18, 21–24]. Two such mechanisms are (1) penetration of the host cuticle by subtilisin- and trypsin-like proteases and (2) protein and lipid uptake using lipases, serine proteases, and zinc-dependent metalloproteases. These may play a similar role as their analogs in ascomycete insect pathogens or fungal pathogens in general. As in other fungal pathogens, powerful genomic and transcriptomic approaches promise to help deconstruct these mechanisms. Three annotated assemblies of genomes in Entomophthoromycotina are currently publicly available, and 52 are in progress. Four studies have investigated the entomophthoralean proteome [25].

## Can pathogens survive without living hosts?

The aforementioned fungal structures are not particularly resistant to environmental extremes. Conidia are moderately resistant, especially thick-walled loricoconidia known in several taxa of *Conidiobolus* and *Pandora*. Loricoconidia occasionally retain the capacity to germinate for several weeks, or even across seasons [26]. To survive unfavorable environmental conditions like winter, entomophthoralean fungi usually produce noninfectious dormant thick-walled resting spores. Pathogens might also survive unfavorable conditions if infected insects become mummified, dormant, or quiescent [27]. Less specialized entomophthoralean pathogens, notably some *Conidiobolus* spp., can survive and grow in the soil. These species often grow well on various nutrient media and can be isolated from soils with no evidence of insect cadavers [28].

## How do entomophthoralean and ascomycete entomopathogens compare for biological control?

In general, infection dynamics operate similarly in Entomophthoromycotina and Ascomycota, often leading to epizootics driven by factors such as host density or weather [29]. However, the two fungal groups utilize host resources differently. Ascomycetous insect pathogens are hemibiotrophs that use the insect as a substrate for nutrition and growth both before (biotrophy) and after (saprotrophy) the insect dies. In contrast, entomophthoralean pathogens are more akin to pure biotrophs, because they first use the resources of the living insect for general nutrition and then sporulate from its cadaver shortly after the insect dies. This second pattern may allow infected insects to be active for longer periods and perhaps even complete a reproductive cycle before dying. Manipulation and applied research of entomophthoralean pathogens for the purpose of biological control can be more difficult due to their slow biotrophic growth, special growth media requirements, and often the high degree of specialization to their hosts. In contrast, ascomycetes (notably biological control agents *Beauveria* and *Metarhizium*) tend to be easier to mass-produce and often have broader host ranges, making them more amenable to development as biopesticides.

Spectacular natural entomophthoralean outbreaks can kill large numbers of pests—often more than ascomycetes. However, only a few entomophthoralean fungi have been successfully manipulated to control harmful insects. Examples include the application of *Zoophthora radicans* to target exotic aphids in Australia [30] and the introduction of *Entomophaga maimaga* to control gypsy moths in Bulgaria [31]. Epizootics caused by *Neozygites* are an integral part of cassava green mite control, allowing farmers to reduce the extent and costs of pesticide use [30]. Thus, while entomophthoralean fungi seldom comprise a “silver bullet” for pest control, their ecological roles in pest mortality and as aids to integrated pest management programs are quite significant. Their fascinating, intimate relationships with a diversity of hosts are well worth further study.

## References

[1] Møller AP. A fungus infecting domestic flies manipulates sexual behaviour of its host. Behav Ecol Sociobiol 1993;33:403–407.

[2] Zurek L, Watson WD, Krasnoff S, Schal C. Effect of the entomopathogenic fungus, *Entomophthora muscae* (Zygomycetes: Entomophthoraceae), on sex pheromone and other cuticular hydrocarbons of the house fly, *Musca domestica*. J Invertebr Pathol 2002;80:171–176. 12384083 10.1016/s0022-2011(02)00109-x

[3] Roy HE, Steinkraus DC, Eilenberg J, Hajek AE, Pell JK. Bizarre interactions and endgames: entomopathogenic fungi and their arthropod hosts. Annu Rev Entomol 2006;51:331–357. doi: 10.1146/annurev.ento.51.110104.150941 16332215 10.1146/annurev.ento.51.110104.150941

[4] Boomsma JJ, Jensen AB, Meyling NV, Eilenberg J. Evolutionary interaction networks of insect pathogenic fungi. Annu Rev Entomol 2014;59:467–485. doi: 10.1146/annurev-ento-011613-162054 24160418 10.1146/annurev-ento-011613-162054

[5] Humber RA. Evolution of entomopathogenicity in fungi. J Invertebr Pathol 2008;98:262–266. doi: 10.1016/j.jip.2008.02.017 18423482 10.1016/j.jip.2008.02.017

[6] Pell JK, Eilenberg J, Hajek AE, Steinkraus DC. Biology, Ecology and Pest Management Potential of Entomophthorales In: Butt TM, Jackson C, Magan N, editors. Fungi as Biocontrol Agents Progress, Problems and Potential. Guildford and King’s Lynn, UK: CABI Publishing; 2001 p. 71–155.

[7] Brobyn PJ, Wilding N. Invasive and developmental processes of *Entomophthora muscae* infecting houseflies (*Musca domestica*). Trans Brit Mycol Soc 1983;80:1–8.

[8] Beauvais A, Latgé J-P. Chitin and β(1–3) glucan synthases in the protoplastic entomophthorales. Arch Microbiol 1989;152:229–236.

[9] Wilding N. *Entomophthora* conidia in the air-spora. J Gen Microbiol 1970;62:149–157. doi: 10.1099/00221287-62-2-149 5493593 10.1099/00221287-62-2-149

[10] Mullens BA, Rodriguez JL, Meyer JA. An epizootical study of *Entomophthora muscae* in muscoid fly populations on southern California poultry facilities, with emphasis on *Musca domestica*. Hilgardia 1987;55:1–41.

[11] Eilenberg J. Abnormal egg-laying behavior of female carrot flies (*Psila rosae*) induced by the fungus *Entomophthora muscae*. Entomol Exp Appl 1987;43:61–65.

[12] Carruthers RI, Larkin TS, Firstencel H, Feng Z. Influence of thermal ecology on the mycosis of a rangeland grasshopper. Ecology 1992;73:190–204.

[13] Watson DW, Mullens BA, Petersen JJ. Behavioral fever response of *Musca domestica* (Diptera: Muscidae) to infection by *Entomophthora muscae* (Zygomycetes: Entomophthorales). J Invertebr Pathol 1993;61:10–16.

[14] Kalsbeek V, Mullens BA, Jespersen JB. Field studies of *Entomophthora* (Zygomycetes: Entomophthorales)—induced behavioral fever in *Musca domestica* (Diptera: Muscidae) in Denmark. Biol Contr 2001;21:264–273.

[15] Hughes DP, Araujo JPM, Loreto RG, Quevillon L, de Bekker C, Evans HC. From so simple a beginning: The evolution of behavioral manipulation by fungi. Adv Gen 2016;94:437–469. 10.1016/bs.adgen.2016.01.004 27131331

[16] Bellini R, Mullens BA, Jespersen JB. Infectivity of two members of the *Entomophthora muscae* complex [Zygomycetes: Entomophthorales] for *Musca domestica* [Dipt.: Muscidae]. Entomophaga 1992;37:11–19.

[17] Boer P. Observations of summit disease in *Formica rufa* Linnaeus, 1761 (Hymenoptera: Formicidae). Myrmecol News 2008;11:63–66.

[18] Małagocka J, Grell MN, Lange L, Eilenberg J, Jensen AB. Transcriptome of an entomophthoralean fungus (*Pandora formicae*) shows molecular machinery adjusted for successful host exploitation and transmission. J Invertebr Pathol 2015;128:47–56. doi: 10.1016/j.jip.2015.05.001 25968105 10.1016/j.jip.2015.05.001

[19] Nair KSS, McEwen FL. *Strongwellsea castrans* (Phycomycetes: Entomophthoraceae), a fungal parasite of the adult cabbage maggot, *Hylemya* (Diptera: Anthomyiidae). J Invertebr Pathol 1973;22:442–449.

[20] Soper RS, Delyzer AJ, Smith LFR. The genus *Massospora* entomopathogenic for cicadas. Part. II. Biology of *Massospora levispora* and its host *Okanagana rimosa*, with notes on *Massospora cicadina* on the periodical cicadas. Ann Entomol Soc Amer 1976;69:89–95.

[21] Xu J, Baldwin D, Kindrachuk C, Hegedus DD. Serine proteases and metalloproteases associated with pathogenesis but not host specificity in the entomophthoralean fungus *Zoophthora radicans*. Can J Microbiol 2006;52:550–559. doi: 10.1139/w06-004 16788723 10.1139/w06-004

[22] Xu J, Baldwin D, Kindrachuk C, Hegedus DD. Comparative EST analysis of a *Zoophthora radicans* isolate derived from *Pieris brassicae* and an isogenic strain adapted to *Plutella xylostella*. Microbiology 2009;155:174–185. doi: 10.1099/mic.0.022103-0 19118358 10.1099/mic.0.022103-0

[23] Grell MN, Jensen AB, Olsen PB, Eilenberg J, Lange L. Secretome of fungus infected aphids documents high pathogen activity and weak host response. Fungal Genet Biol 2011;48:343–352. doi: 10.1016/j.fgb.2010.12.003 21156213 10.1016/j.fgb.2010.12.003

[24] Freimoser FM, Screen S, Hu G, St Leger R. EST analysis of genes expressed by the zygomycete pathogen *Conidiobolus coronatus* during growth on insect cuticle. Microbiology 2003;149:1893–1900. doi: 10.1099/mic.0.26252-0 12855740 10.1099/mic.0.26252-0

[25] de Fine Licht H, Hajek AE, Jensen AB, Eilenberg J. Utilizing genomics to study entomopathogenicity in the fungal phylum Entomophthoromycota: A review of current genetic resources. Adv Genet 2016;94:42–64. 10.1016/bs.adgen.2016.01.003 27131322

[26] Brobyn PJ, Wilding N, Clark SJ. The persistence of infectivity of conidia of the aphid pathogen *Erynia neoaphidis* on leaves in the field. Ann Appl Biol 1985;107:365–376.

[27] Eilenberg J, Thomsen L, Jensen A. A third way for entomophthoralean fungi to survive the winter: slow disease transmission between individuals of the hibernating host. Insects 2013;4:392–403. doi: 10.3390/insects4030392 26462426 10.3390/insects4030392 PMC4553471

[28] Manning RJ, Walters SD, Callaghan AA Saprotrophy of *Conidiobolus* and *Basidiobolus* in leaf litter. Mycol Res 2007;111(2):1437–1449. 18035528 10.1016/j.mycres.2007.08.019

[29] Hesketh H, Roy HE, Eilenberg J, Pell JK, Hails RS. Challenges in modeling complexity of fungal entomopathogens in semi-natural populations of insects. BioControl 2009;55:55–73.

[30] Nielsen C, Wraight SP. Exotic aphid control with pathogens In: Hajek AE, Glare TR, O'Callaghan M, editors. Use of microbes for control and eradication of invasive arthropods: Springer 2009 pp. 90–113.

[31] Georgiev G, Mirchev P, Rossnev B, Petkov P, Georgieva M, Pilarska D, et al Potential of *Entomophaga maimaiga* Humber, Shimazu and Soper (Entomphthorales) for suppressing *Lymantria dispar* (Linnaeus) outbreaks in Bulgaria. Compt Rend Acad Bulg Sci 2013;66:1025–1032.

